# Evaluating Users’ Emotional Experience in Mobile Libraries: An Emotional Model Based on the Pleasure-Arousal-Dominance Emotion Model and the Five Factor Model

**DOI:** 10.3389/fpsyg.2022.942198

**Published:** 2022-07-05

**Authors:** Yang Zhao, Dan Xie, Ruoxin Zhou, Ning Wang, Bin Yang

**Affiliations:** ^1^School of Information Management, Wuhan University, Wuhan, China; ^2^School of Information Technology & Management, University of International Business and Economics, Beijing, China

**Keywords:** emotional experience, UX, PAD, FFM, mobile library

## Abstract

As a part of user experience, user emotion has rarely been studied in mobile libraries. Specifically, with the proposed emotional model in combination with the Pleasure-Arousal-Dominance (PAD) Emotion Model and the Five Factor Model (FFM), we evaluate user emotions on the mobile library’s three IS features (i.e., user interface, interaction quality, and service environment). An experience procedure with three tasks has been designed to collect data. 50 participants were enrolled, and they were asked to fill in questionnaires right after the experience. The correlations among the PAD emotions were examined. Specifically, users have a low perception of pleasure (P), high perception of arousal (A), and low perception of dominance (D). However, these three emotional states were not always significantly correlated with each other. This study extends mobile library research by focusing on users’ emotional experience. Specifically, the detailed PAD emotions have been examined. This study provides a new approach for application developers and managers to evaluate the user experience of an application.

## Introduction

In the past decade, to provide better services, libraries have begun to provide mobile services (Zhao et al., 2015). In this study, mobile library refers to using mobile terminals and mobile networks to provide services, including book retrieval and borrowing, library seat reservation, library notices, and other information services. As a primary platform for people in universities and research institutes to get access to information, mobile libraries have received extensive attention. Nowadays, most mobile libraries have already satisfied people’s basic needs to access information through mobile devices anytime and anywhere. However, with people gradually taking the good usability and convenience of mobile libraries for granted, they have higher expectations for mobile library services. Among these expectations, user experience is of the most importance. In recent years, researchers have explored the factors affecting user experience in mobile library (e.g., Li et al., 2015; Wang et al., 2018).

In the 1990s, the term “user experience (UX)” was popularized by Donald Norman, a leader in the Human Computer Interaction (HCI) community. In traditional economy, UX is driven by commodities, goods, and services that are external to the buyer and often ignores their inherently personal needs. With the rise of the experience economy, UX is more driven by users’ internal needs, including their emotional, physical, intellectual and even spiritual needs (Pine and Gilmore, 1998). People do not necessarily have to do something even if they are able to. Willingness is important, and it is where emotions can come into play.

Mobile library studies have shown that user emotion generated in the interactions will influence user experience, and then affect user satisfaction and usage continuance (Qiao, 2017; Shen and Zheng, 2019). For example, Lopatovska (2014) has proposed a framework to study the influence of emotions in information search process. Also, Barifah and Landoni (2020) have studied digital library users’ emotions elicited in the search process and indicated that the emotional effect is a significant factor in user experience. Given the importance of emotion in user experience, to engage users, application designs should make them “happy.” Driven by user needs, instead of focusing on the practical and functional aspects, UX researchers extend their research to users’ emotional experience. If developers wish to design information systems that users enjoy, they must have the ability to recognize, understand, and adapt to users’ emotions. As an application of information systems, it is necessary for mobile libraries to capture users’ emotional states after adoption to provide users with better services and to improve users’ satisfaction.

When using mobile library services, several IS features may affect users’ emotional experience. From the interactive perspective, we examine how user interface, interaction quality, and service environment influence mobile library users’ emotion. An approach is proposed to measure users’ emotional states accurately by collecting data right after using the mobile library application. Besides measuring users’ emotion with the Pleasure-Arousal-Dominance (PAD) Emotion Model, we take personality traits into account. Research has shown that users’ emotional states are also influenced by their personality traits (Wang et al., 2022). Emotional states are temporary and have time dependencies. For example, Wei et al. (2020) indicated that current emotional states were affected not only by the situation, but also by previous emotional trajectories. Therefore, our study uses the PAD Emotion Model and the Five Factor Model (FFM) to represent users’ emotions and personality traits respectively.

In this study, we have recruited 50 participants to experience a mobile library application and to complete three tasks. The participants were required to first fill in the demographic Big Five Inventory (BFI) and then fill in the PAD emotion scale as soon as they have completed the whole task. The advantage of asking users to fill in the PAD emotion scale right after the application usage is that it helps to avoid the inaccuracy brought by obtaining data by retrospection. In previous studies, surveys and/or interviews were mainly conducted well after the participants’ experience to collect data rather than during the active decision-making process. Specifically, participants answered the questionnaires by recalling their experiences in the past (Venkatesh et al., 2003), which is inaccurate. This study examines the model by using the data collected right after the participants complete all tasks and log out of the mobile library application, which ensures the accuracy of responses. This study has the following potential contributions to the field. First, by focusing on users’ emotional experience and integrating the PAD Emotion Model and the FFM, which has been rarely examined in the field of mobile library, this study extended mobile library research by emphasizing the importance of user emotion. Second, this study underlines the role of different interactive IS features in influencing users’ emotional experience in mobile libraries. Third, this study provides a new approach for application developers and managers to evaluate users’ emotional experience when they use an application. Finally, this study overcomes the limitation of obtaining data by retrospection.

The paper includes seven sections. Section 2 is the literature on mobile library, PAD Emotion Model, the FFM, and the IS features that affect user experience. Section 3 proposes the model and the formulas of the emotional model. Detailed research methods, data analysis, and discussion are presented in Section 4 to Section 6. Finally, Section 7 concludes the theoretical and practical implications, and limitations.

## Literature Review

### Mobile Library

Mobile libraries, supported by mobile technology, play a key role in providing library services. In the mobile era, people are used to fragmentary reading (Zhao et al., 2016). For now, fundamental functions of mobile libraries are well developed. From the survey results of Ma and Dong (2020), many universities provide mobile libraries. Among the investigated subjects, 22 universities provide library mobile information services by Wireless Application Protocol (WAP) and 35 universities provide these services by applications. With technological advancement, people can acquire information anytime and anywhere with little effort.

Digital libraries mainly aims to provide abundant e-books and rich electronic resources (Huang et al., 2015; Ye et al., 2020). Studies on digital libraries were mainly about how to construct special resource databases, the functions of digital library, the extension of service patterns, and the evaluation (Wang, 2013; Xu and Du, 2018; Li and Liu, 2019). In the wave of mobile Internet, mobile libraries provide intelligent services, such as QR code (Zhao et al., 2015) and WeChat mobile library services (Liu, 2021). However, with people getting used to mobile library applications, these functions can no longer satisfy users’ advanced needs. Nowadays, mobile library developers are more concerned about how to improve user experience, instead of just providing basic functions. Therefore, improving the UX of mobile libraries has drawn researchers’ attention.

Extant literature on UX of mobile libraries mainly explored users’ perception of technology usage and esthetic feeling (Lai et al., 2014; Ke and Su, 2018; Wang et al., 2018; Xu and Guo, 2018). These researchers mainly evaluated UX from the function (i.e., the quality of system, information and service), the visual design (i.e., the hierarchical structure, the elements, and the colors), and the facilities (i.e., the wireless network and the internet of things) of mobile libraries, which are utilitarian and objective. However, mobile library users’ emotional experience has rarely been examined. Some researchers have considered user emotion as one of the indicators affecting general UX (Qiao, 2017; Chen, 2020), but how IT features affect mobile library users’ emotional experience is scarce. Different from extant literature, we investigate how interactive IT features affect users’ emotional experience by measuring users’ emotional states right after using a mobile library application.

### Interactive IS Features That Affect Mobile Library Users’ Emotional Experience

The emotional experience is part of UX, which is elicited through users’ interaction with the product/system. Many studies have provided evidence that interactive IS features can affect users’ emotional experience. For example, from the perspective of user-interface interaction, researchers have found that website design could affect users’ emotional states (Molinillo et al., 2021).

In this study, we focus on the influence of interactive IS features. In specific, UX arose from an interaction between an individual and a system. The interaction aims at accomplishing a particular task, takes place in a certain context and lasts for a limited period, and is an iterative process (Fu and Inskip, 2019; Yang et al., 2019). In the APEC (Esthetic, Practical, Emotional, and Cognitive) model proposed by Vyas and Van Der Veer (2005), interaction is regarded as a key factor to explain the users’ actual experience after using the application. To categorize these interactive IS features, we divide the interaction into three parts: user-interface, user-others and user-environment.

We have the following three interactive IS features. First, as for the user-interface interaction, we examine the design of the “user interface.” Nielsen (1997) proposed that website developers should retain users by providing a highly usable interface. Also, in a certain context, features of the product/system – such as functionality and interface design – affect the interaction. Mobile libraries aim at keeping users active in the application, where its interface plays a key role in users’ emotional experience. Second, “interaction quality” is used to examine the influence of interaction between users and others. Interaction quality looked at the way users interact with each other online in a constructive manner (Zhou et al., 2019). As direct mobile library service providers, librarians’ attitudes and professionalism have a direct influence on service performance when users ask for help (Zhao, 2014; Xu and Zhang, 2021), which affects users’ emotional experience. Also, as a new e-learning platform (Zhao et al., 2015), mobile libraries allow users to exchange information and share ideas with their friends. Whether the sharing ways are quick and convenient will also affect users’ emotional experience. Third, regarding the user-environment interaction, we focus on the “service environment”. The survey results of Brade et al. (2017) indicated that UX is environmentally dependent. UX of mobile applications is affected by mobile devices, mobile service systems, and mobile network environments. Also, the importance of different environment context in users’ experience of personal mobile products has been emphasized by Korhonen et al. (2010). To summarize, this study considers three interactive IS dimensions influencing mobile library users’ emotional experience: (1) user interface, (2) interaction quality, and (3) service environment.

### Measure Emotional Experience With the Pleasure-Arousal-Dominance (PAD) Emotion Model

To investigate users’ emotional experience when they use mobile libraries, we adopt the PAD Emotion Model. First, emotion has been rarely examined in the field of mobile library. This study focuses on the influence of three interactive IS features on mobile library users’ emotional experience. Second, we use the PAD Emotion Model to measure users’ emotional states. PAD Emotion Model outperforms other emotional measurements as it not only covers multiple emotional dimensions but also has a strong theoretical foundation.

**FIGURE 1:**
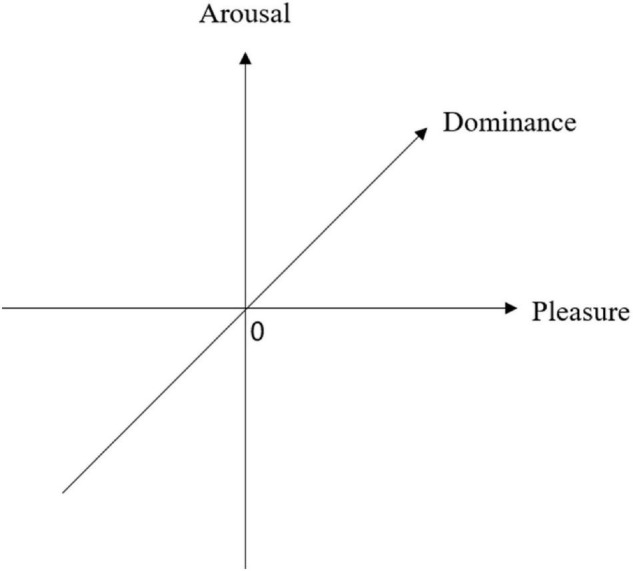
The Pleasure-Arousal-Dominance (PAD) Emotion Model.

In the PAD Emotion Model, *Pleasure* ranges from extreme unhappiness to extreme happiness. *Arousal* is conceived as a feeling ranging from sleep to frantic excitement. *Dominance* ranges from submissiveness to dominance (Mehrabian and Russell, 1974). They developed three orthogonal scales of emotion: pleasure, arousal, and dominance (see Figure 1).

Mehrabian and Russell (1974) developed the corresponding emotional measurement tool (i.e., the PAD emotion scale). The PAD Emotion Model reduces the implementation difficulty of emotional measurement and improves the accuracy and efficiency of emotional measurement. Based on the initial orthogonal axes, there are eight combinations of P, A, and D. Table 1 presents the label of the diagonals of the PAD space. In specific, Mehrabian and Russel used +P to represent pleasure (and −P for displeasure), +A to represent arousal (and −A for non-arousal), and +D to represent dominance (and −D for submissiveness).

**TABLE 1:** Emotions in the three-dimensional Pleasure-Arousal-Dominance (PAD) space^1^.

Exuberant (+P+A+D)	Bored (–P–A–D)
Dependent (+P+A–D)	Disdainful (–P–A+D)
Relaxed (+P–A+D)	Anxious (–P+A–D)
Docile (+P–A–D)	Hostile (–P+A+D)

*^1^This is adopted from Mehrabian (1997).*

### Emotional Model With Personality Traits

When studying user emotional experience, personality traits are important factors that should be considered. Previous studies have provided evidence that personality trait could be used in recognizing personalized emotion (Zhao et al., 2019) and will affect user experience (Zheng et al., 2022). Kshirsagar (2002) developed a multilevel model of emotion that incorporates personality traits into the emotional experience and links personality to emotional transitions in case studies. Based on a dimensional view of emotion, Guo (2008) established an intensity-based personalized emotion model by taking personality traits as an emotional component.

To measure personality traits, we adopt the widely used Five Factor Model (FFM) (Zuckerman, 1994). The FFM provides a taxonomy of personality. This model uses personality dimensions to indicate different psychological functions. Further, each function consists of more concrete features (Zhao and Seibert, 2006).

The FFM include *Openness to Experience*, *Conscientiousness*, *Extraversion*, *Agreeableness*, and *Neuroticism* (*OCEAN*). *Openness to Experience* describes people with curiosity and loves new experiences. *Conscientiousness* measures how motivated, persistent and arduous an individual is in goal pursuit. *Extraversion* characterizes the degree of assertiveness, enthusiasm and etc. *Agreeableness* assesses one’s interpersonal orientation. *Neuroticism* measures the ability to keep emotional stability. The descriptions are shown in Table 2.

**TABLE 2:** The description of each factor in the Five Factor Model (FFM).

Five factors	Description
Openness to Experience	Creative, innovative, imaginative, reflective, untraditional
Conscientiousness	Diligent, persistent
Extraversion	Cheerful
Agreeableness	Cooperative, modest
Neuroticism	Anxiety, hostility, depression, self-consciousness, impulsiveness

People’s personalities can be represented by numerical scores of these five factors. Specifically, the larger the value of a certain factor, the more obvious the individual’s performance on this factor.

### Emotional Model Based on the Pleasure-Arousal-Dominance (PAD) Emotion Model and the Five Factor Model (FFM)

In combination with the PAD Emotion Model and the FFM, this section proposes an emotional model. As discussed, the distinctiveness of emotional states strongly depends on the personality of the focal person. Therefore, we adopt the FFM to measure users’ personality traits. The emotional states of users are determined by the PAD Emotion Model.

The mapping from the OCEAN to the three-dimensional PAD space is defined as follows (Gebhard, 2005):

(1) Pleasure = 0.21 * Extraversion + 0.59 * Agreeableness + 0.19 * Neuroticism,
(2) Arousal = 0.15 * Openness + 0.3 * Agreeableness – 0.57 * Neuroticism,
(3) Dominance = 0.25 * Openness + 0.17 * Conscientiousness + 0.6 * Extraversion −0.32 * Agreeableness.

Gebhard (2005) used the mapping to calculate an individual’s default mood and verified that it was suitable for defining people’s personalities. Using time series methods, Mehraei and Akcay (2017) adopted the mapping to measure the initial mood of individuals when predicting the P, A, and D. Results show that all PAD can be predicted.

We define the personality traits that are mapped from the OCEAN traits into the three-dimensional PAD space as P_0_, A_0_, D_0_, and the pleasure, arousal, and dominance scores obtained from the PAD emotion scale as P_1_, A_1_, D_1_. In this research, the formulas of the emotional model are as follows:

(1) P Score = P_1_ − P_0_,
(2) A Score = A_1_ − A_0_,
(3) D Score = D_1_ − D_0_.

## Methodology

This section provides the design details, including the material, participants, measures, and procedures. Overall, people’s personalities can be represented by numerical scores of these five factors. Specifically, the larger the value of a certain factor, the more obvious the individual’s performance on this factor. To capture user emotion given the three IS features under study, we designed an experience procedure with three tasks (examining the three IS features respectively). In this study, 50 students were recruited to experience the given mobile library application and to finish the three tasks. Participants were asked to fill in the Big Five Inventory (BFI) before they began the experience and then they were required to complete three tasks. To ensure that data were collected timely and could reflect users’ actual emotion on each IS feature, participants were asked to fill in the PAD emotion scale right after they have finished each task. The goal of task one is to collect the participants’ P1, A1, D1 about the user interface; task two aims to collect the participants’ P1, A1, D1 about the interaction quality; and the goal of task three is to collect the participants’ P1, A1, D1 about the service environment of the mobile library application.

### Material

**FIGURE 2:**
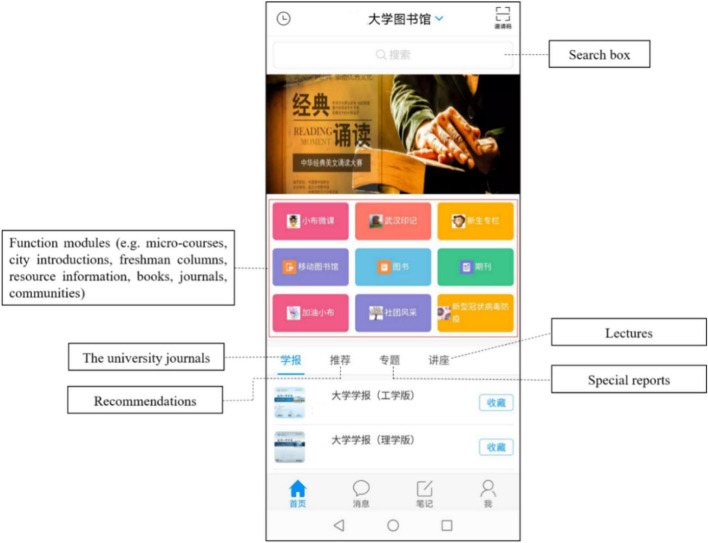
Homepage of the university mobile library application.

**FIGURE 3:**
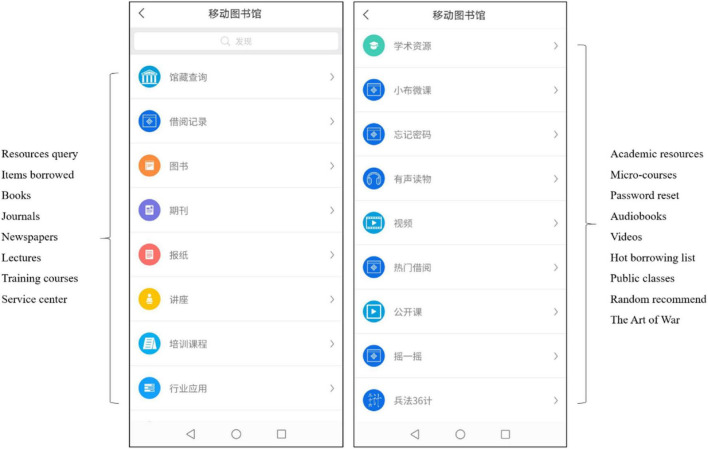
Resource information modules of the university mobile library application.

A University mobile library application in China (see Figures 2, 3) where users search for books, reserve books to borrow, reserve seats to study, and get notices of the latest book arrival, was used as the material in the experience procedure. The application has been launched for years and is open to users in different occupations, which is ideal for the research context.

To control the influence of the mobile device system fluency, participants were asked to use the smartphones (Xiaomi 9) provided by the research team.

### Participants

Given our research purpose, we recruited 50 students who volunteered to take part in the experience procedure by posting the recruitment notice on the BBS of the university. Nielsen (1992) has pointed out that we can find 80% of the problems on the website by testing 5 users and 95% of the problems by testing 10 users. The larger the sample size is, the more reliable the results will be. Specifically, among the 50 recruited participants, 25 male students and 25 female students participated in the procedure during 2019.8.6–2019.8.30 in the library of this university. After finishing the whole procedure, the participants would receive gifts in turn.

### Measures

Big Five Inventory (BFI), a 5-point Likert Scale that constitutes of short phrases, is used to obtain the OCEAN traits of users (John and Srivastava, 1999). In this study, the participants are Chinese. Thus, this research used a Chinese simplified edition of the PAD emotion scale to obtain users’ emotional states. This version, which is a 9-point Likert Scale with 12 pairs of opposite adjectives, is a modification of the Mehrabian PAD emotion scale for Chinese users, and its validity has been examined (Li, 2008).

### Procedure

The whole experience procedure was conducted through three steps:

**Step 1. Introduction to the experience procedure**

Introduce the purpose and process of the experience procedure to participants at the beginning. This introduction part lasted about 5 min.

**Step 2. Fill in the BFI**

Guide the participants to fill in the demographic Big Five Inventory (John et al., 1991). This course lasted about 5 min.

**Step 3. Complete experience tasks**

Task One: The participants were asked to log in to the application and browse the interface, including page layout, color, search box, and navigation bar. Each participant was required to browse the interface for at least 2 min and then fill in the PAD emotion scale.

Task Two: The participants were asked to search the book “The old man and the sea” only once. If found, then, they were asked to share it with friends in their social networks or other users on the platform. Otherwise, they could turn to the librarian online for help. Besides, the participants were required to notice whether there were interactive ways like the message, voice, picture, video, and interactive channels like QQ, WeChat, microblog, e-mail when they would like to share the searching results with others. When they finished, they were requested to fill in the PAD emotion scale.

Task Three: The participants were asked to search and download the designated essay (the authors are Lo Patrick et al., and the published journal in Library Hi Tech). Through this task, they were able to experience the response speed of opening a new page, the stability, and the security of the mobile network. When they finished, they were requested to fill in the PAD emotion scale.

The duration time of each task was 5–10 min, and each task aimed to collect the participants’ P1, A1, and D1 about the user interface, the interaction quality, and the service environment of the university library application.

## Data Analysis

### Data Pre-Processing

This step targets at standardizing the data obtained from the Big Five Inventory and the Chinese simplified version of the PAD emotion scale. With the obtained OCEAN traits of participants, we use the mapping in section 3 to transform the OCEAN traits into the three-dimensional PAD space. In addition, we calculate the pleasure, arousal, and dominance scores of participants from the Chinese simplified version of the PAD emotion scale. Specifically,^1^

(1) pleasure trait = (Q1 + Q4 + Q7 + Q10)/4,
(2) arousal trait = (Q2 + Q5 + Q8 + Q11)/4,
(3) dominance trait = (Q3 + Q6 + Q9 + Q12)/4.

After the standardization, we can calculate the data in the same coordinate system.

Table 3 presents the demographic information. 78% of participants were in the 21–26 age groups, which conforms to the status (Zhao et al., 2015).

**TABLE 3:** Demographic data of the participants.

Demographics	Item	Frequency	Percentage (%)
Gender	Male	25	50
	Female	25	50
Age	18–20	6	12
	21–23	21	42
	24–26	18	36
	>26	5	10
Education	Undergraduate	25	50
	Graduate or above	25	50
Have you ever	Yes	39	78
used the app?	No	11	22

### Model Analysis

**FIGURE 4:**
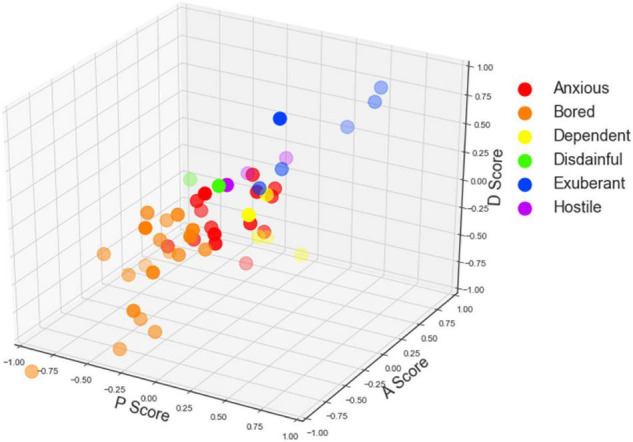
The scatter plot of participants’ Pleasure-Arousal-Dominance (PAD) scores of the user interface (For Figures 4–6, the *x*-axis represents the pleasure score, the *y*-axis represents the arousal score, and the *z*-axis represents the dominance score. The colors represent different emotion categories).

**FIGURE 5:**
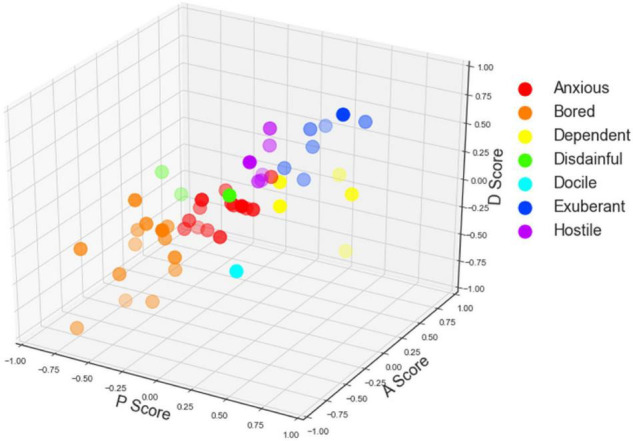
The scatter plot of participants’ Pleasure-Arousal-Dominance (PAD) scores of the interaction quality.

**FIGURE 6:**
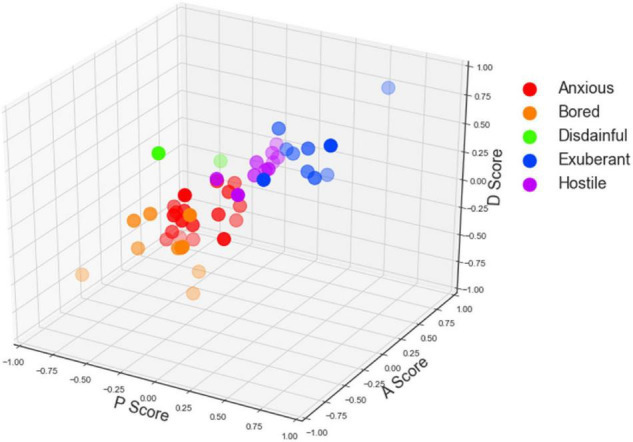
The scatter plot of participants’ Pleasure-Arousal-Dominance (PAD) scores of the service environment.

With the PAD emotional model, we calculate the pleasure, arousal, and dominance scores regarding the user interface, the interaction quality, and the service environment of the mobile library application. The three-dimensional scatters are shown in Figures 4–6. In specific, Figures 4–6 displays participants’ perceptions of pleasure, arousal, and dominance of the application’s interface, interaction, and service, respectively.

According to Table 1, participants’ emotions can be classified into eight categories by +P versus −P, +A versus −A, and +D versus −D. With the data obtained in task one, participants’ emotions of the user interface of the mobile library application were classified into six categories (i.e., anxious, bored, dependent, disdainful, exuberant, and hostile). With the data obtained in task two, participants’ emotions of the interaction quality of the mobile library application were classified into seven categories (anxious, bored, dependent, disdainful, docile, exuberant, and hostile). With the data obtained in task three, participants’ emotions of the service environment of the mobile library application were classified into five categories (anxious, bored, disdainful, exuberant, and hostile).

As shown in Figures 4–6, the scatters mostly gather in the corner where the arousal score is positive, but the pleasure and dominance scores are negative. The result indicates that most participants feel anxious and bored with the mobile library application. Participants’ perceptions of pleasure and dominance are low whereas their perceptions of arousal are high. The user interface, the interaction quality, and the service environment of the mobile library application can activate the physiological activity of users and keep them concentrated. However, it fails to give users positive emotional feedback and high self-control.

Significantly, in Figure 6, there are more scatters in the upper right than those in the left bottom, which means that the total number of good emotion categories in Figure 6 is more than that in Figures 4, 5. The result indicates that participants’ emotional states of the service environment are more positive than those of the user interface and the interaction quality. Users’ experience of the service environment of the mobile library application is better than the other two dimensions.

### Correlation Analysis

From the scatters above, we find that after the three tasks, participants’ perceptions of arousal were always high, whereas their perceptions of pleasure and dominance were always low. To explore the relationship between the scores about these three IS features, we conduct the Pearson correlation analysis among P-A-D Scores of the user interface, the interaction quality, and the service environment. With the correlation analysis, we could examine: (1) if a certain perceived emotion is consistent in different IS features; and (2) given a specific IS feature, if the P-A-D scores are correlated with each other.

Table 4 summarizes the correlation results. First, the correlations between each emotion score are examined across different IS features. That is, we look into the P, A and D scores respectively. We found that, P Score of the user interface, the interaction quality and the service environment are significantly correlated with each other. However, A Score of the user interface is insignificantly correlated with A Score of the service environment, and D Score of the user interface is insignificantly correlated with D Score of the service environment either. This shows that regarding each emotional dimension, Pleasure appears to be step-dependent, whereas Arousal and Dominance seem to be more independent. Second, we investigate the relationship between P/A/D in each IS feature. The results indicate that in all these three IS features, P Score, A Score and D Score are significantly correlated with each other. That is, users’ perceptions about pleasure, arousal and dominance are consistent in each IS feature.

**TABLE 4:** Correlation among P-A-D Scores of the user interface, the interaction quality, and the service environment.

		User interface			Interaction quality			Service environment		
				
		P Score	A Score	D Score	P Score	A Score	D Score	P Score	A Score	D Score
User interface	P Score	1								
	A Score	0.803**	1							
	D Score	0.673**	0.715**	1						
Interaction quality	P Score	0.591**	0.532**	0.330*	1					
	A Score	0.544**	0.657**	0.378**	0.811**	1				
	D Score	0.389**	0.396**	0.412**	0.613**	0.641**	1			
Service environment	P Score	0.298*	0.226	0.164	0.605**	0.525**	0.402**	1		
	A Score	0.067	0.237	0.052	0.342*	0.464**	0.258	0.725**	1	
	D Score	0.127	0.078	0.186	0.314*	0.233	0.387**	0.715**	0.631**	1

** p < 0.01; * p < 0.05.

## Discussion

This study has used an emotional model with personality traits to examine users’ emotional experience in mobile libraries regarding three IS features, and the main findings are as follows. First, from the emotional model, we found that most users felt anxious and bored after using the mobile library application, their perceptions of arousal were high whereas their perceptions of pleasure and dominance were low. The arousal traits reflect users’ physiological activity levels. That is, users were highly focused when using this mobile library application, which indicated the satisfying design of the user interface, the interaction quality, and the service environment of this mobile library application. The pleasure traits reflect users’ happiness while the dominance traits reflect users’ control over the environment. Most users were at a low level of happiness and control over the application, which means that the user interface, interaction quality and service environment of the mobile library application cannot bring users happiness, and users are restricted in the use process.

Second, with the emotion scores, we could evaluate this mobile application’s user interface, interaction quality, and service environment. In specific, user interface reflects the esthetics and convenience of the web design, including the layout, readability, color scheme, font size, line spacing, logos, navigation bar and multimedia (Walia et al., 2016; Cheung et al., 2017; Koranteng et al., 2021; Liu, 2022; Zhao and Huang, 2022). This study shows that the esthetics and convenience of the mobile library application have done a good job attracting users’ attention, but they failed to provide users with a happy experience. Developers should pay more attention to identifying users’ emotional needs and identify users’ preferences, so as to design products that can make users more delighted. Interaction quality measures the social function of the application, including sharing channels, sharing ways, and customer service response (Chessa and Solari, 2021). We found that although the mobile library application provides social functions, the sharing channels and sharing ways were too limited to meet users’ social demands. The slow response speed of the librarians also hindered users from getting pleasure and dominance when they ask for help. Service environment reflects the usability and function of the application, including mobile network speed, application loading speed, application stability and application security (Wei and Yang, 2017; Wang et al., 2018; Wei et al., 2018; Xu and Guo, 2018). The result demonstrates that the usability of the mobile library application performed better than the user interface and the interaction quality, but it still failed to provide users with pleasure and dominance. When the mobile network is slow and the application is unstable, users will lose patience and happiness and feel controlled by the network and the system.

Finally, the results show that perceptions of pleasure about the user interface, interaction quality and service environment are significantly correlated with each other. Also, users’ perceptions of arousal about the interaction quality are correlated with those about the user interface and service environment. However, users’ perceptions of arousal about the user interface and service environment are not correlated with each other. Also, users’ perceptions of dominance about the interaction quality are correlated with those about the user interface and service environment, but the correlation relationship between perceptions of dominance about user interface and service environment is insignificant. The results pointed out that the interaction quality of the mobile library is critical in improving users’ emotional experience, which confirms the finding of Zhao (2014) that the interaction quality of mobile services reflected the overall service level in the multi-dimensional interaction between users and mobile libraries and determined UX. In addition, results of the correlation analysis verified that users’ perceptions of PAD emotions are interdependent, which is consistent with previous findings (Chang et al., 2014; Miniero et al., 2014; Liu et al., 2016; Verkijika and De, 2019). Specifically, the effect of arousal on pleasure is positive. That is, people will feel happy when an event brings them excitement. Also, the influence of dominance on pleasure is positive. When people have control over the environment, they are more satisfied.

## Conclusion

### Theoretical Contributions

In this study, the PAD Emotion Model and the FFM are combined to develop the emotional model, and the three IS features influencing users’ emotional experience are used as different dimensions in mobile libraries. The PAD Emotion Model is used to determine the emotional states after using the mobile library application, whereas the FFM model is used for identifying the personality traits of users. The potential theoretical and managerial implications are as follows.

First, by focusing on users’ emotional experience and integrating the PAD Emotion Model and the FFM, this study investigated the rarely examined topic and extended mobile library research. Prior research mainly focused on how objective system design features affect UX (Vila et al., 2021), neglecting the role of user emotion. This study emphasized the importance of user emotion, with the circumstance that mobile library functions are mature and UX becomes more important. It advances mobile library research by emphasizing the importance of user emotion.

Second, this study provides a new approach for application developers and managers to evaluate users’ emotional experience when they use an application. The emotional model proposed by this study took users’ personality traits into consideration with the FFM, which controls the influence of personality traits on emotions. Since more and more application users have diverse cultural backgrounds and values with the globalization of trade, collecting information about users’ personality traits could help applications provide better services.

Third, this study overcomes the limitation of obtaining data by retrospection. To measure users’ emotional states accurately, we collect data right after participants use the mobile library application. For researchers, they can design an experience procedure when they have to collect real-time responses. For application developers and managers, taking advantage of information technology, they can invite users to fill out questionnaires right after an experience. For example, pop-up window could be used to collect real-time information.

### Practical Implications

First, the interactions between user-interface (interface design), user-others (interaction design) and user-environment (environment optimization) are equally important. In specific, when designing the interface, mobile library developers are suggested to improve the functionality, the navigation and the visualization of the interface to provide users with better experience. As for the interaction, social elements could be added in the mobile library application to encourage interactions. Besides sharing books and other content, in-app chatroom or discussion forum could be developed to engage users. Regarding the environment, developers are suggested to enhance the infrastructure to provide users with smooth experience.

Second, user emotion should be considered when designing the interaction of mobile libraries. To increased pleasure, mobile library developers could enhance visual design with proper color scheme, improve usage fluency by optimizing the performance of different infrastructure components, etc. To get user aroused, developers could innovatively design story lines to get users involved. Besides, arousal levels could be increased with ambient design elements like background music. To help users feel dominant, navigation should be clear and easy to use. Also, services should be accessible so that users could feel everything is under control.

Third, in combination with big data analytics, mobile libraries could profile their users. Thus, their personality traits could be used. Further, as suggested by the study, real-time feedback is much better than retrospection. Rather than sending questionnaires, real-time responses could be acquired by pop-up feedback window. With this design, users could voice their dissatisfaction and get timely solutions. Thus, users may feel more pleasant and be satisfied with the mobile library application.

### Limitations

First, this study only chose a specific university library application as the material of experience procedure, but future research may consider other forms of mobile libraries such as WAP and WeChat. Second, the participants are a small group of students in this university, and faculties are not included. Future research may diversify the participant characteristics to explore the influence of different perceptions, such as the esthetics, interaction modes and search habits. Finally, although this study has examined the relationships among the perceptions of PAD emotions about the user interface, the interaction quality and the service environment, the causal relationships among these three emotions were not examined. Future research may propose hypotheses and construct a theoretical model to explore their causal relationships.

## Data Availability Statement

The original contributions presented in the study are included in the article/supplementary material, further inquiries can be directed to the corresponding author.

## Author Contributions

YZ and BY: conceptualization. BY: methodology. RZ, NW, and DX: validation. BY: formal analysis. RZ and BY: writing—original draft preparation. RZ and DX: writing—review and editing. BY and NW: visualization. YZ: supervision, project administration, and funding acquisition. All authors have read and agreed to the published version of the manuscript.

## Conflict of Interest

The authors declare that the research was conducted in the absence of any commercial or financial relationships that could be construed as a potential conflict of interest.

## Publisher’s Note

All claims expressed in this article are solely those of the authors and do not necessarily represent those of their affiliated organizations, or those of the publisher, the editors and the reviewers. Any product that may be evaluated in this article, or claim that may be made by its manufacturer, is not guaranteed or endorsed by the publisher.

## Footnotes

1 In the Chinese simplified version of the PAD emotion scale, Q1, Q4, Q7, and Q10 are adjectives to assess pleasure of the participants, Q2, Q5, Q8, and Q11 are adjectives to assess arousal of the participants, Q3, Q6, Q9, and Q12 are adjectives to assess dominance of the participants (see Appendix A).

## Appendix

**Appendix A: Chinese simplified version of the Pleasure-Arousal-Dominance (PAD) emotion scale:** The scale is a semantic differential scale, consisting of four items for each PAD components. The 9-point Likert scale constitutes of pairwise adjectives. The higher the score is, the higher your perception of pleasure, arousal and dominance is.

	−4	−3	−2	−1	0	1	2	3	4	
Q1. Annoyed										Pleased
Q2. Sleepy										Wide-awake
Q3. Controlled										Controlling
Q4. Melancholic										Contented
Q5. Calm										Excited
Q6. Submissive										Dominant
Q7. Despairing										Hopeful
Q8. Relaxed										Stimulated
Q9. Awed										Important
Q10. Unsatisfied										Satisfied
Q11. Sluggish										Frenzied
Q12. Influenced										Influential

*The original PAD emotion scale used in this study was Chinese version, the translation cited here is the adjectives that Mehrabian and Russell (1974) had used to evaluate PAD emotions.*
